# The Antioxidant, Anti-Inflammatory and Moisturizing Effects of *Camellia oleifera* Oil and Its Potential Applications

**DOI:** 10.3390/molecules29081864

**Published:** 2024-04-19

**Authors:** Lijun Zhou, Yunlan Peng, Zhou Xu, Jingyi Chen, Ningbo Zhang, Tao Liang, Tao Chen, Yao Xiao, Shiling Feng, Chunbang Ding

**Affiliations:** 1 College of Life Science, Sichuan Agricultural University, Ya’an 625014, China; zhouzhou124@126.com (L.Z.); 18982957389@163.com (Y.P.); 18380465781@139.com (J.C.); 13363580763@163.com (N.Z.); lxltlqh@163.com (T.L.); chentao293@163.com (T.C.); 72120@sicau.edu.cn (Y.X.); 2 Panxi Crops Research and Utilization Key Laboratory of Sichuan Province, Xichang University, Xichang 615000, China; xzhbiol@163.com

**Keywords:** *Camellia oleifera* oil, antioxidants, moisturizing, anti-inflammatory, emulsion

## Abstract

*Camellia oleifera* oil (CO oil) extracted from *C. oleifera* seeds has a 2300-year consumption history in China. However, there is relatively little research regarding its non-edible uses. This study determined the physicochemical properties of CO oil extracted via direct pressing, identified its main components using GC-MS, and evaluated its antioxidant, moisturizing, and anti-inflammatory activities. The results revealed that CO oil’s acid, peroxide, iodine, and saponification values were 1.06 ± 0.031 mg/g, 0.24 ± 0.01 g/100 g, 65.14 ± 8.22 g/100 g, and 180.41 ± 5.60 mg/g, respectively. CO oil’s tocopherol, polyphenol, and squalene contents were 82.21 ± 9.07 mg/kg, 181.37 ± 3.76 mg/kg, and 53.39 ± 6.58 mg/kg, respectively; its unsaturated fatty acid (UFA) content was 87.44%, and its saturated fatty acid (SFA) content was 12.56%. CO oil also demonstrated excellent moisture retention properties, anti-inflammatory effects, and certain free radical scavenging. A highly stable CO oil emulsion with competent microbiological detection was developed using formulation optimization. Using CO oil in the emulsion significantly improved the formulation’s antioxidant and moisturizing properties compared with those of the emulsion formulation that did not include CO oil. The prepared emulsion was not cytotoxic to cells and could reduce cells’ NO content; therefore, it may have potential nutritional value in medicine and cosmetics.

## 1. Introduction

*Camellia oleifera* Abe1., an evergreen tree of the Theaceae genus *Camellia*, is native to China, where it has been cultivated for 2300 years [1]. The oil extracted from its mature seeds is a premium health and wellness oil known as ‘Oriental olive oil’. *Camellia oleifera* oil (CO oil) is rich in oleic acid, linoleic acid, α-linolenic acid, stearic acid, vitamin E, squalene, phytosterols, and triterpenoids. Vitamin E, polyphenols, and flavonoids have been shown to have good antioxidant activity [2,3,4]. “The Supplements of the Compendium Materia Medica”, “The Chinese Pharmacopoeia”, and “The Dictionary of Chinese Medicine” all list CO oil as a traditional Chinese medicine. CO oil has received widespread attention and application in the food and health industries.

In 2023, China’s Camellia oleifera planting area was approximately 4.67 × 10^4^ km^2^, and its CO oil production capacity was expected to reach one million tons. It is estimated that by 2025 China’s Camellia oleifera planting area will be more than 6 × 10^4^ km^2^ square meters, and that its CO oil production capacity will increase to 2 million tons. Although CO oil is recognized as a high-grade health care oil compared with the most widely used rapeseed oil, the price of edible oil of the same volume is at least ten times higher, making it difficult for tea oil to be fully promoted and applied.

In ancient times, CO oil was used to care for hair and make beauty creams, and was directly applied to affected areas to treat burns and eczema. As people’s understanding of CO oil’s nutritional benefits continues to increase, multiple industries, including the food, medicine, and cosmetics industries, have facilitated related product innovations. Some hair care and beauty essential oils are starting to use tea oil as one of their raw materials. It is popular to extract skin-beneficial elements from natural plants for use in cosmetics and to create new green products [5]. Utilizing oil from natural plants as a base for creating cosmetics has a solid track record. The study and development of olive oil and grape seed oil have significantly expanded the use of natural oil resources in cosmetics [6]. 

The oil-in-water (O/W) emulsification method is commonly used in cosmetics formulations for the market because of its affinity with the skin [7]. This study evaluated CO oil’s basic efficacy and physical and chemical properties, applied CO oil to moisturizing lotion, and evaluated the antioxidant, moisturizing, and anti-inflammatory activities of CO oil and lotion with and without CO oil to provide a reference for comprehensive CO oil utilization.

## 2. Results and Discussion

### 2.1. CO Oil Qualities

CO oil’s chemical properties and functional active compounds were evaluated. CO oil’s acid, peroxide, iodine, and saponification values were found to be 1.06 ± 0.031 mg/g, 0.24 ± 0.01 g/100 g, 65.14 ± 8.22 g/100 g, and 180.41 ± 5.60 mg/g, respectively. These acid and peroxide levels were equal to those of the optimal quality index of China’s national standard (GB/T 11765-2018) [8]. The acid value indicates the amount of free fatty acids in an oil, whereas the peroxidation value indicates its degree of fatty acid oxidation. The iodine content reflects the degree of unsaturation in organic compounds and is a measure of an oil’s storage stability. The iodine content was below 100 g/100 g, suggesting that the oil was non-drying [9]. The saponification value reveals an oil’s fatty acid molecular weight [10]. Overall, CO oil’s basic physicochemical properties were found to be superior to China’s standards, and thus sufficient to support subsequent experiments.

Natural antioxidants in plants, such as tocopherols, polyphenols, and squalene, are thought to break long chains or alter lipid free radicals during free chain reactions, thereby enhancing vegetable oil’s antioxidant properties. CO oil’s tocopherol, polyphenol, and squalene contents were 82.21 ± 9.07 mg/kg, 181.37 ± 3.76 mg/kg, and 53.39 ± 6.58 mg/kg, respectively. Plant polyphenols play a role in antioxidant, anti-inflammatory, immune response stimulation, and detoxification processes [11]. Squalene, the main component of skin surface polyunsaturated lipids, nurtures the skin as an emollient and antioxidant, and has hydrating and antitumor properties [12]. Tocopherols are the most prevalent antioxidants in cosmetics [13] and have been shown to be helpful in reducing erythema generated by acute UV radiation exposure edema, sunburn, photoaging, immunosuppression caused by solar radiation, and even skin cancer [14,15]. 

**Figure 1:**
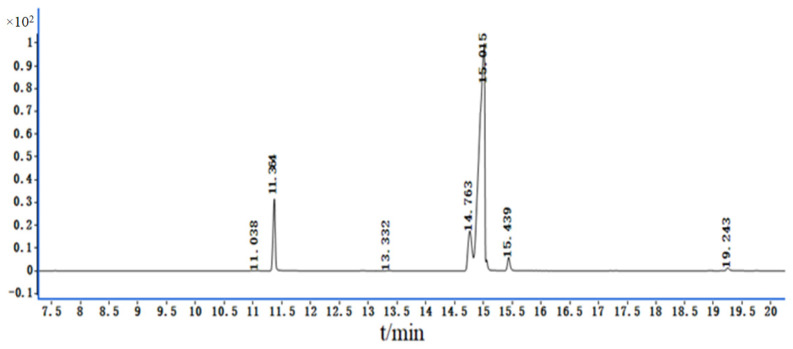
The total ion flow diagram of fatty acid composition in CO oil.

As shown in Figure 1 and Table 1, CO oil’s fatty acid composition includes palmitoleic acid (C16:1), palmitic acid (C16:0), linoleic acid (C18:2), oleic acid (C18:1), stearic acid (C18:0), and eicosenoic acid (C20:1), but does not include erucic or trans fatty acids, which are harmful to human health. CO oil’s content included 87.44% unsaturated fatty acids (UFAs) and 12.56% saturated fatty acids (SFAs). According to these results, CO oil’s UFA content was approximately seven times higher than its SFA content. This means that, when CO oil acts on human skin, it forms a protective film on the skin’s surface to prevent water loss and provides a large quantity of UFAs that cannot be synthesized by the human body, thus exhibiting anti-inflammatory, anti-allergic, and other effects [16,17]. 

### 2.2. In Vitro Antioxidant Ability Analysis of CO Oil and Emulsion

**Figure 2:**
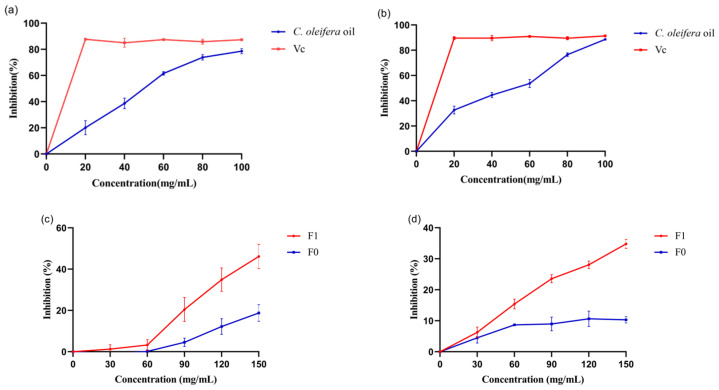
The antioxidant ability of (**a**) CO oil on scavenging DPPH^•^, (**b**) CO oil on scavenging ABTS^•+^, (**c**) emulsion on scavenging DPPH^•^ and (**d**) emulsion on scavenging ABTS^•+^. Note: F0 means emulsion without CO oil, F1 means emulsion with CO oil.

Assays using 2,2-Diphenyl-1-picryl-hydrazyl (DPPH) and 2,2-Azino-bis-(3-ethylbenzothiazole-6-sulphonic acid) (ABTS) were selected to test the antioxidant capacities of CO oil and emulsion formulations. As shown in Figure 2, CO oil and emulsion formulations exhibited a concentration-dependent DPPH radical-scavenging effect. When the CO oil concentration reached 100 mg/mL, DPPH’s radical-scavenging activity reached 78.61%. The IC50 was 53.48 mg/mL. At this concentration, CO oil’s ABTS-scavenging ability was equivalent to that of Vc. The IC50 was 46.45 mg/mL. This CO oil’s radical-scavenging action may be attributable to the nature of phenolics, which add to their electron transfer (hydrogen-donating) capacity [18,19]. According to a previous study, sesamin extracted from CO oil may also attach to cell membranes and defend against free radical attack [20]. Moreover, CO oil’s oxidant stability is comparable to that of sesame oil, as CO oil contains components with potent antioxidant properties, such as polar phenol and vitamin E [21]. Therefore, adding CO oil to the emulsion may have neutralized free radicals in skin cells. When the concentrations of F0 and F1 reached 150 mg/mL, the DPPH radical-scavenging activity reached 46.15% and 18.75%, respectively. ABTS radical-scavenging activities followed the same trend as DPPH radical scavenging did, and there were only slight differences in the results. At all the concentrations examined, the emulsion without CO oil (F0) demonstrated low antioxidant activity. Interestingly, when the concentration was 150 mg/mL, the DPPH^•^ and ABTS^•+^ clearance rates of the emulsion containing CO oil increased by approximately 27.4% and 25.0%, respectively, compared with those for the emulsion without CO oil. The emulsion consisted of 4.0% CO oil, which means it contained 6 mg/mL of CO oil. According to the antioxidant activity map, CO oil’s antioxidant activity was less than 10% at this concentration. Therefore, it can be considered that the antioxidant activity of emulsion 1 added with 6 mg/mL CO oil was much higher than that of CO oil with the same concentration. The application of oil in the oil-in-water emulsion improved the emulsion’s antioxidant activity. It is speculated that these significantly increased activities were not entirely due to the addition of CO oil, but may be due to the enhanced antioxidant activity of CO oil after emulsification, or the synergistic effects of various components after CO oil was added. 

### 2.3. Analysis of Moisturizing Ability of CO Oil and Emulsion

**Figure 3:**
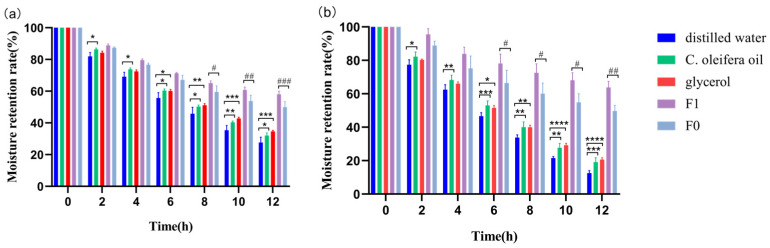
The moisture retention rate of (**a**) CO oil and two emulsions at 80% relative humidity, and (**b**) CO oil and two emulsions at 43% relative humidity. (n = 3 * *p* < 0.05; ** *p* < 0.01; *** *p* < 0.001; **** *p* < 0.0001; # *p* < 0.05; ## *p* <0.01; ### *p* < 0.001). Note: F0 means emulsion without CO oil; F1 means emulsion with CO oil.

Glycerol has a beneficial moisturizing effect in cosmetics [22]. As depicted in Figure 3, after 12 h, both CO oil, two emulsions, and glycerol had more outstanding moisturizing capabilities than the vehicle (deionized water) group in an 81% atmosphere and 43% relative humidity, and there was no significant difference between the moisture retention rates of glycerol and CO oil. The moisturizing effect of the emulsion was significantly better than the effects of CO oil and glycerin, and the emulsion’s continuous moisturizing ability was stronger. This may have been because the emulsion contained more chemicals with moisturizing qualities than pure CO oil did, which interacted with CO oil to produce a more effective moisturizing effect. The human skin provides a barrier capable of regulating temperature and controlling excessive or inadequate moisture [23]. Hydrating the skin prevents skin cells from losing moisture through evaporation. CO oil is rich in unsaturated fatty acids. When CO oil is applied to the skin, it forms an oily membrane barrier that inhibits water loss from skin cells, achieving the desired moisturizing effect [24]. F1’s moisture retention was significantly superior to that of F0. The results showed that adding CO oil to cosmetics could significantly improve cosmetic products’ moisturizing abilities. On the one hand, using CO oil strengthens the emulsion’s water-locking impact on the skin. To a certain extent, the emulsion forms a barrier on the skin to prevent water loss due to external factors [25]. On the other hand, its working period proportionally increases and the moisturizing effect lasts longer, resulting in a higher moisturizing value.

### 2.4. Analysis of Anti-Inflammatory Capacities of CO Oil and Emulsion

**Figure 4:**
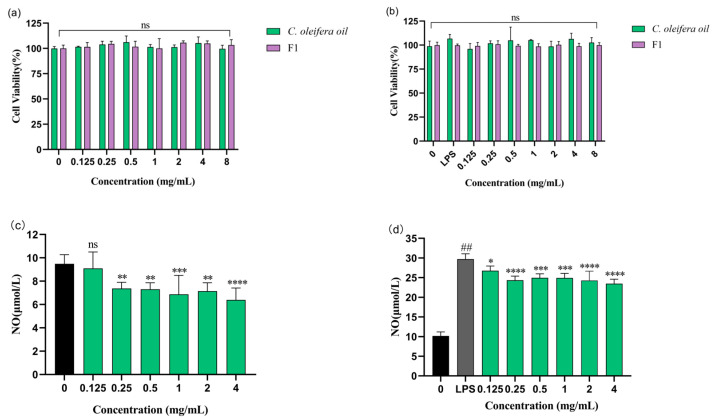
Effect of CO oil and F1 on the cell viability. (**a**) RAW 264.7 cells were incubated with various concentrations of CO oil or F1 for 24 h. (**b**) RAW 264.7 cell were co-cultured with LPS and CO oil or F1 for 24 h. (**c**) RAW 264.7 cells were cultured with CO oil for 24 h and NO secretion was measured by the Griess reagent. (**d**) RAW 264.7 cells were cultured with CO oil with LPS stimulation for 24 h. LPS-stimulated NO secretion was measured by the Griess reagent. Values are means ± SD; n = 5. Note: ns means not significant. F0 means emulsion without CO oil; F1 means emulsion with CO oil. ## means comparison with blank control *p* < 0.0001; * means comparison with LPS-stimulated *p* < 0.05; ** *p* < 0.01; *** *p* < 0.001; **** *p* < 0.0001.

The cytotoxic effects of CO oil and emulsions containing CO oil (F1) on RAW 264.7 cells were detected via a CCK-8 assay. As shown in Figure 4, neither CO oil nor emulsions containing CO oil affected the viability of RAW 264.7 cells at 0.125–8 mg/mL. CO oil was thus characterized as a safe and healthy plant oil that can be used as a base oil for cosmetic products. To avoid cytotoxicity induced by the co-treatment of lipopolysaccharide (LPS) and CO oil, which could affect the assessment of anti-inflammatory ability, the cell activity of the LPS and CO oil co-culture or the emulsion containing CO oil was determined. The results showed that in the LPS that was co-cultured with CO oil and in the emulsion containing CO oil, cell activity had no significant indigenous effect. Therefore, LPS could be used as an inducer in the anti-inflammatory experiment, as it has no significant effect on the cells themselves [26]. NO is an endogenous gaseous signal molecule that can produce a type of inflammatory mediator via macrophage when macrophages were stimulated by LPS [27]. We conducted subsequent experiments based on this principle. 

As shown in Figure 4c, spontaneous NO secretions were significantly decreased by CO oil incubation. CO oil inhibited NO by 4.13, 22.21, 22.97, 27.45, 24.66, and 32.59% at doses of 0.125, 0.25, 0.5, 1.0, 2.0, and 4.0 mg/mL, respectively. Its inhibitory effect gradually increased as the CO oil concentration increased. At the same time, the inhibition rate was above 20% when the CO oil concentration reached 0.25 mg/mL or higher. This effect was significant. Figure 4d shows the results of a comparison with the control group; the amount of NO increased 2.93 times in the LPS-induced group. CO oil inhibited LPS-induced NO by 9.91, 18.10, 15.97, 16.14, 18.26, and 20.98% at doses of 0.125, 0.25, 0.5, 1.0, 2.0, and 4.0 mg/mL, respectively. These results indicate that CO oil can effectively prevent and inhibit inflammatory reactions in human skin cells when used as a basic cosmetic ingredient, thus reducing the incidence of skin allergy symptoms, because it is a pure, natural and healthy plant oil. In folk remedies, CO oil is commonly applied to burns for anti-inflammatory purposes. According to these results, it can be inferred that this practice has a certain basis in fact.

**Figure 5:**
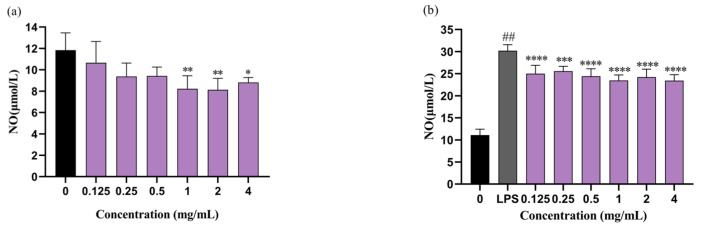
(**a**) RAW 264.7 cells were cultured with F1 for 24 h and NO secretion was measured by the Griess reagent. (**b**) RAW 264.7 cells were cultured with F1 with LPS stimulation for 24 h. LPS-stimulated NO secretion was measured by the Griess reagent. Values are means ± SD, n = 5. Note: ns means not significant. Values are means ± SD; n = 5. F0 means emulsion without CO oil; F1 means emulsion with CO oil. ## means comparison with blank control *p* < 0.0001; * means comparison with LPS-stimulated *p* < 0.05; ** *p* < 0.01; *** *p* < 0.001; **** *p* < 0.0001.

As shown in Figure 5, F1 inhibited NO by 30.53, 31.33, and 25.52% at doses of 1.0, 2.0, and 4.0 mg/mL, respectively (Figure 4c). LPS treatment alone significantly enhanced NO production (30.20 μM) compared to the control group (11.10 μM), whereas F1 inhibited LPS-induced NO by 17.16, 15.38, 19.09, 22.28, 19.82, and 22.46% at doses of 0.125, 0.25, 0.5, 1.0, 2.0, and 4.0 mg/mL, respectively (Figure 5b). The results showed that the emulsion had an inhibitory effect on both co-cultured NO and NO produced spontaneously, thus alleviating the inflammatory response to a certain extent. Additionally, the anti-inflammatory activity in the emulsion could still be reflected, indicating that CO oil was the source of this effect. 

### 2.5. Optimization of Emulsion Formulation

Based on the fundamental formula presented in Table 2, the various impacts of octyldodecanol lauroyl glutamate, PEG-100 glycerol monostearate, cetyl ethylhexanoate, CO oil, glycerol, and xanthan gum on the emulsion were investigated in detail. Each of the six aforementioned factors was considered at three distinct levels. The orthogonal test was conducted using the L18 (3^7^) design table presented as Table A1. The No. 5 emulsion exhibited the highest dispersion effect, absorption, skin sensation, and centrifugal stability. According to the table, octyldodecanol lauroyl glutamate, glycerol, PEG-100 glycerol monostearate, xanthan gum, cetyl ethylhexanoate, and CO oil were the emulsion’s primary and secondary components. The optimal mixture comprised 1.0% octyldodecanol lauroyl glutamate, 1.0% PEG-100 glycerol monostearate, 4.0% CO oil, 1.0% cetyl ethylhexanoate, 6.0% glycerol, and 0.3% xanthan gum. The final formulation is shown in Table 2.

### 2.6. Stability Tests

After the emulsion was manufactured, its stability was assessed, and it was subjected to a variety of stability tests over 28 days. Throughout the investigation, its color and odor remained essentially unaltered. The emulsion was pale yellow and odorless because the formula contained no additional additives. No phase separation or granules were noticed during testing, indicating that the emulsion maintained homogeneity and appearance. During the investigation, the emulsion’s pH ranged from 5.74 to 6.03. The human skin’s pH ranges between 4.5 and 6. Thus, the emulsion’s pH range was optimal. The sample’s sensory qualities, appearance, and homogeneity were not altered, thus proving that the emulsion was stable and the product’s development was satisfactory.

### 2.7. Microorganism Detection

Through the measurement of total bacterial counts, including counts of thermotolerant coliform bacteria, staphylococcus aureus, fungi, yeasts, and pseudomonas aeruginosa, the emulsion’s microorganism content was determined to be acceptable. This finding may be attributable to the presence of preservatives in the formula, demonstrating that the anti-corrosion properties of the formula were sufficient.

## 3. Materials and Methods

### 3.1. Materials

Squalane, grape seed oil, isopropyl myristate (IPM), dimethyl silicone oil (DC200), vitamin E, Hansen gum, hyaluronic acid (HA), glycerin, and butylene were obtained from Guangzhou Baiyu Biotechnology Co., Ltd., (Guangzhou, China). Methanol, trichloromethane, glacial acetic acid, potassium iodide, sodium thiosulfate, nitric acid, sulfuric acid ethanol, sodium chloride, isopropanol, and potassium hydroxide were purchased from Chengdu Kelong Chemical Plant (Chengdu, China). The Cell Counting Kit-8 (CCK-8) was purchased from Boster Biological Technology Co., Ltd., (Wuhan, China). DMEM high glucose medium and fetal bovine serum were purchased from Hyclone (Logan, UT, USA).

### 3.2. Oil Extraction and Determination Assay

#### 3.2.1. Oil Extraction Assay

*C. oleifera* seeds were obtained from Perfume Village, Maohe Town, Mingshan District, Ya’an City, in 2021 (Ya’an, China). *C. oleifera* seeds were dried to a constant weight in a 60 °C oven. CO oil was extracted via physical pressing.

#### 3.2.2. Determination of Acid, Peroxide, Saponification, and Iodine Values

The acid, peroxide, iodine, and saponification values were determined according to international standards (ISO 660-2009, ISO 3960-2007, ISO 3657-2013, and ISO 3961-2018, respectively) [28,29,30,31]. 

### 3.3. Determination of Tocopherol

Tocopherol levels were determined using methods from previous studies [32] with slight modifications. A weight of 1 g of oil was dissolved in 10 mL of n-hexane and evenly mixed. Samples were filtered through a 0.45 μm microporous membrane and analyzed using an Agilent 1260 HPLC (Agilent Technologies, Santa Clara, CA, USA) equipped with a ZORBAX SB-C18 column (4.6 mm × 150 mm, 5.0 μm), and a fluorescence detector. A 295 nm excitation wavelength and 325 nm emission wavelength were used. The methanol mobile phase used a 0.8 mL/min flow rate and a 35 °C column temperature.

### 3.4. Determination of Polyphenols

Polyphenols were extracted following a modified version of the previous method [2]. A weight of 0.5 g of oil was dissolved in 5 mL of n-hexane. Next, the sample was subjected to three extractions using 2 mL of aqueous methanol (80%, *v*/*v*) each time. The combined extract was left at room temperature (25 °C) overnight. The residual oil was washed using n-hexane. Methanol was added to bring the volume to 10 mL for polyphenol content determination. To determine the polyphenol content, 0.1 mL of the polyphenol extract was mixed with 0.02 mL of Folin–Ciocalteu solution for 5 min. Next, 0.08 mL of 10% sodium carbonate and 0.8 mL of distilled water were added. After incubation in the dark for 1 h, the mixture’s absorbance was measured at 765 nm using distilled water as a blank and a Spectra Max M2 microplate reader.

### 3.5. Determination of Squalene

The previous method was used to extract squalene, and 0.2 g of oil was treated using a 2 mL 2 mol/L potassium hydroxide/ethanol solution [33]. The samples were mixed and saponified at 80 °C for 60 min. After cooling it to room temperature (25 °C), the sample was subjected to three extractions using 2 mL of distilled water and 5 mL of n-hexane each time. The n-hexane phase was further washed with distilled water until it reached a neutral pH. A concentrated solution was obtained using a rotary evaporator. Subsequently, samples were analyzed using an Agilent 1260 high-performance liquid chromatograph. The analysis was performed using a ZORBAX SB-C18 column (4.6 mm × 150 mm, 5.0 μm), a diode array detector, a 210 nm detection wavelength, and a 30 °C column temperature. The mobile phase consisted of methanol/acetonitrile (60/40 *v*/*v*), and the flow rate was maintained at 1.0 mL/min.

### 3.6. Determination of Fatty Acid Composition

Fatty acid methyl esters (FAMEs) were determined using the potassium hydroxide/methanol method [34]. Approximately 100 mg of CO oil was placed in a mill glass plug test tube and treated with 2 mL of 1 mol/L NaOH/methanol. The sample was thoroughly mixed using a vortex mixer and shaken at 40 °C for 30 min. The methyl ester was extracted using 2 mL of n-hexane, and the aqueous phase was discarded. The n-hexane extract was washed with distilled water, dried with anhydrous sodium sulfate, and analyzed using GC-MS after filtration. 

GC-MS analysis of the FAMEs was performed using an Agilent 7890A gas chromatograph and a 5977C mass spectrometer. The capillary column was an HP-5MS (30 m × 0.25 mm; 0.25 μm). The temperature program consisted of an initial hold at 80 °C for 1 min, followed by an increase to 160 °C at a rate of 20 °C/min for 1 min, a further increase to 200 °C at 20 °C/min for 1 min, and a final increase to 250 °C at 5 °C/min for 5 min. Helium was the carrier gas, and we used a flow rate of 0.6 mL/min and a split ratio of 50:1. The ion source was EI, with a mass scan range of 50–500 *m*/*z* and a solvent delay of 3 min. The FAME profiles were identified by comparing them with the National Institute of Standards and Technology Library (NIST) database. The individual fatty acid content is expressed as a percentage of the total fatty acid content [35].

### 3.7. Determination of Antioxidant Activities

#### 3.7.1. DPPH Radical-Scavenging Assay

The DPPH free radical-scavenging assay for samples was determined according to previous work with slight modifications [36]. Briefly, a 70 μL test sample solution or VC solution as a standard solution was mixed with 140 μL of 0.1 mmol/L DPPH solution. The reaction was carried out in the dark for 30 min at room temperature (25 °C). The absorbance was measured at 517 nm using a 96-well microplate. The scavenging ability was calculated using the following equation:

Inhibition (%) = (A_0_ − A_1_)/A_0_ × 100

where A_0_ is the absorbance of the DPPH solution without a sample, and A_1_ is the absorbance of the DPPH solution with a sample. 

#### 3.7.2. ABTS Radical Scavenging Assay

The ABTS radical-scavenging assay was evaluated according to previous work [37], with minor modifications. The ABTS radical cation (ABTS^•+^) was generated by reacting a 7.4 mmol/L ABTS stock solution with 2.6 mmol/L potassium persulfate and leaving the mixture in the dark at room temperature (24–26 °C) for 12 to 16 h before use. Ethanol was used to dilute the ABTS^•+^ solution to an absorbance of 0.70 ± 0.01 at 734 nm. Next, 100 μL of the sample was mixed with 100 μL of ABTS, followed by a 6 min incubation period. The absorbance was measured at 734 nm. The scavenging ability was calculated using the following equation:

Inhibition (%) = (A_0_ − A_1_)/A_0_ × 100

where A_0_ is the absorbance of the ABTS solution without a sample, and A_1_ is the absorbance of the ABTS solution with a sample.

### 3.8. Moisturizing Assay

Moisture retention and moisture absorption tests were performed using methods modified from previous studies [38]. A 1 g sample was placed in a dryer at 25 ± 1 °C and a relative humidity of 43% (potash) or 80% (ammonium sulfate). The sample’s weight was recorded at 0, 2, 4, 6, 8, 10, and 12 h. The test sample’s moisture retention ability was evaluated using the percentage of residual water in the test sample:

Retention rate (%) = (W_t_/W_0_) × 100

where W_0_ is the sample’s weight before desiccation (g), and W_t_ is the sample’s weight after the designated time in the desiccator at 25 °C (g).

### 3.9. Anti-Inflammatory Assay

#### 3.9.1. Cell Culture

RAW 264.7 murine macrophage cells were kindly provided by the Stem Cell Bank, Chinese Academy of Sciences, Shanghai, China. RAW 264.7 cells were cultured in Dulbecco’s modified eagle medium (DMEM) containing 10% FBS at 37 °C in a humidified atmosphere of 95% air and 5% CO_2_.

#### 3.9.2. Cell Viability Assay

The viability of the RAW 264.7 cell sample was determined using a CCK-8 assay. Cells (1 × 10^5^ cells/mL) were planted in a 96-well microplate and maintained at 37 °C for 6 h before various concentrations of the sample were added. After 24 h of treatment, the solutions were completely replaced with fresh new medium. Next, 10 µL of CCK-8 was added into the wells and the plate was placed at 37 °C for 30 min before the absorbance was read at 450 nm. The inhibition rate was calculated according to the following formula:

Inhibition rate (%) = A_1_/A_0_ × 100

where A_0_ was the absorbance value of the blank group and A_1_ was the absorbance value of the sample treatment group.

#### 3.9.3. Determination of Nitric Oxide (NO)

NO production was detected using Griess reagent [39]. Cells (1 × 10^5^ cells/mL) were planted in a 96-well microplate and maintained at 37 °C for 6 h. After attaching, cells were pretreated with different sample concentrations for 2 h, and then, the LPS (5 µg/mL) was added for co-treatment for 24 h. The supernatant was then mixed with Griess reagent and incubated for 10 min in the dark. The absorbance was measured at 540 nm. The NO content was calculated using a nitrite standard curve.

### 3.10. Emulsion Optimization

#### 3.10.1. Emulsion Preparation

The emulsion formulation was based on information obtained from previous studies [40,41] and is shown in Table 2. First, the A-phase and B-phase were weighed according to the formulation. Certain amounts of A and B were heated to 85 °C; A’s ingredients were prepared, mixed with B, and stirred until completely dissolved. The sample was stirred for 30 min and cooled to 65 °C. Next, C was added to the previous mixture, which was then well mixed and maintained at 65 °C for 20 min. The sample was then removed and cooled at room temperature for one night.

#### 3.10.2. Experimental Development of Emulsion Formulation

The emulsion’s base formulation was optimized using orthogonal testing. Emulsions were comparatively evaluated for various parameters, including feeling, absorption, dispersion, and centrifugation evaluation, to select the best emulsion base formulation for further study. The specific emulsion evaluation standard is shown in Table 3.

### 3.11. pH Measurement

A 1.0 g sample was added to 9.0 g of ultrapure water and stirred well. Next, the mixed solution was heated in a water bath to 40 °C and then cooled to 25 °C. The formulations’ pHs were measured using a pH meter (PHS-3E, Shanghai LeiMag Instrument Co., Ltd., Shanghai, China). 

### 3.12. Stability Testing

Heat resistance, cold resistance, and centrifuge tests were evaluated using methods described in a previous study [42]. 

Centrifugation experiment: The CO oil emulsion was centrifuged at 4000 r/min for 30 min to check whether the samples stratified. 

Heat resistance test: The CO oil emulsion was placed in a 40 °C environment for 28 days. Samples were checked for delamination after they returned to room temperature (25 °C). 

Cold resistance test: The CO oil emulsion was placed in a −4 °C environment for 28 days. Samples were checked for stratification after they returned to room temperature (25 °C).

### 3.13. Detection of Microorganisms

Bacteriological tests were performed according to generally accepted hygienic standards for cosmetics.

### 3.14. Statistical Analysis

Data were statistically analyzed via two-way ANOVA using GraphPad Prism 8 (GraphPad Software, Inc., La Jolla, CA, USA). All values are given as the mean ± standard deviation. Every experiment was conducted in triplicate. A *p*-value less than 0.05 was deemed statistically significant.

## 4. Conclusions

CO oil’s antioxidant capacity was determined using DPPH and ABTS assays, which returned values of 78.61 ± 1.55% of DPPH and 88.62 ± 0.78% of ABTS. CO oil’s in vitro moisturizing capacity was comparable with that of glycerin. The developed emulsion formulation was optimized to have excellent stability and certified microbiological detection. The inclusion of CO oil can significantly improve an emulsion’s antioxidant and in vitro moisture retention capacities compared with those of an emulsion formulation without CO oil. The CO oil and prepared emulsion had no cytotoxicity to cells and could reduce cells’ NO content. This demonstrated that CO oil has excellent skin protection properties and is ideal for cosmetics production and use. 

## Figures and Tables

**Table 1:** Results of fatty acid GC-MS analysis.

Number	Compound	Retention Time/min	Relative Content/%
1	palmitoleic acid	11.038	0.053
2	palmitic acid	11.364	9.83
3	linoleic acid	14.763	9.75
4	oleic acid	15.015	77.64
5	stearic acid	15.439	2.14
6	eicosenoic acid	19.243	0.60

**Table 2:** The formula of CO oil emulsion.

Phases	Material	Property	Amount (%)	
			F1	F0
A	Octyl dodecanol lauroyl glutamate	Emulsifier	1.0	1.0
	PEG-100 glycerol monostearate	Emulsifier	1.0	1.0
	Cetyl eehylhexanoate	Softener	1.0	1.0
	Isopropyl mylistate	Softener	3.0	3.0
	Dimethicone	Softener	2.0	2.0
	CO oil	Softener	4.0	
	Vitis vinifera (Grape) seed oil	Antioxidant	1.0	1.0
	Tocopherol	Antioxidant	0.5	0.5
B	xanthan gum	Thickening agent	0.3	0.3
	Sodium Hyaluronate	Humectant	0.1	0.1
	Glycerin	Humectant	6.0	6.0
	Butanediol	Solvent	2.0	2.0
	deionized water	Solvent	77.3	81.3
C	Polyol preservatives		0.8	0.8

Note: F0 is the emulsion without CO oil and F1 is the emulsion contain CO oil.

**Table 3:** Emulsion evaluation standard table.

Index	Scores			
	Excellent (20–25)	Good (15–20)	Qualified (10–15)	Poor (Less than 10)
dispersion	very good smear, strong liquidity	easy to smear, liquidity qualified	difficult to smear, a little sticky	not good smear, poor liquidity
absorption	fast absorption	fast absorption but a little oil floating on the skin surface	the absorption rate is a bit slow	the absorption rate is slow and a large amount of oil floats on the skin surface
feeling	the skin feels very refreshing but not greasy	the skin feels refreshing	a little greasy	the skin feels very greasy
centrifugation	uniform and delicate appearance without change	non-stratified form	there is a slight stratification	stratification

## Data Availability

Data are contained within the article.

## Appendix A

**Table A1:** Orthogonal test results of formula.

Number	Octyl Dodecanol Lau-Royl Glutamate/%	PEG-100 Glycerol Monostearate/%	CO Oil/%	Cetyl Eehylhexanoate/%	Glycerin/%	Xanthan Gum/%	Aggregate Score
1	0.5	0.5	3.0	0.5	4.0	0.1	78.2
2	0.5	1.0	4.0	1.0	5.0	0.2	86.6
3	0.5	1.5	5.0	1.5	6.0	0.3	78.7
4	1.0	0.5	4.0	0.5	5.0	0.3	88.3
5	1.0	1.0	5.0	1.0	6.0	0.1	91.7
6	1.0	1.5	3.0	1.5	4.0	0.2	83.7
7	1.5	0.5	3.0	1.0	6.0	0.2	79.9
8	1.5	1.0	4.0	1.5	6.0	0.3	80.4
9	1.5	1.5	5.0	0.5	5.0	0.1	77.4
10	0.5	0.5	5.0	1.0	4.0	0.3	76.9
11	0.5	1.0	3.0	0.5	6.0	0.3	85.3
12	0.5	1.5	4.0	1.0	4.0	0.1	69.5
13	1.0	0.5	5.0	1.0	4.0	0.3	79.4
14	1.0	1.0	3.0	1.5	5.0	0.1	81.9
15	1.0	1.5	4.0	0.5	4.0	0.1	82.2
16	1.5	0.5	4.0	1.5	6.0	0.1	80.6
17	1.5	1.0	5.0	0.5	4.0	0.2	77.3
18	1.5	1.5	3.0	1.0	5.0	0.3	82.9
K1	79.2	80.5	81.5	82.0	78.1	79.8	
K2	84.5	83.9	81.7	81.2	82.3	81.1	
K3	79.6	79.0	80.2	80.2	82.9	82.5	
R	5.3	4.8	1.5	1.8	4.7	2.7	
